# Women’s perceived social support: associations with postpartum weight retention, health behaviors and depressive symptoms

**DOI:** 10.1186/s12905-019-0839-6

**Published:** 2019-11-21

**Authors:** Sabrina Faleschini, Lynne Millar, Sheryl L. Rifas-Shiman, Helen Skouteris, Marie-France Hivert, Emily Oken

**Affiliations:** 1 School of Psychology, Laval University, Quebec, QC Canada; 2 Australian Health Policy Collaboration, Victoria University, Melbourne, VIC Australia; 3 Australian Institute for Musculoskeletal Science, The University of Melbourne and Western Health, St Albans, Melbourne, VIC Australia; 4 Division of Chronic Disease Research Across the Lifecourse, Department of Population Medicine, Harvard Medical School and Harvard Pilgrim Health Care Institute, Boston, MA USA; 5 Monash Centre for Health Research Implementation, School of Public Health and Preventive Medicine, Monash University, Clayton, VIC Australia; 6 Department of Nutrition, Harvard T.H. Chan School of Public Health, Boston, MA USA

**Keywords:** Health behaviors, Depression, Postpartum, Social support, Body weight changes

## Abstract

**Background:**

Social support may promote healthful behaviors that prevent excess weight at critical periods in women’s life. Our objective was to investigate associations of social support at 6 months postpartum with women’s health behaviors that have previously been shown to predict weight retention at 1 year postpartum.

**Methods:**

At 6 months postpartum in Project Viva, a pre-birth prospective cohort in Massachusetts, women reported social support using the Turner Support Scale, depressive symptoms using the Edinburgh Postnatal Depression Scale, diet using PrimeScreen, average number of hours walking, light/moderate and vigorous physical activity, television viewing, and sleeping each day.

**Results:**

Among 1356 women, greater partner support was associated with higher levels of walking (OR 1.36, 95% CI [1.01, 1.82]) and intake of fiber (OR 1.43, 95% CI [1.06, 1.91]) and lower intake of trans-fat (OR 1.49, 95% CI [1.11, 2.01]). Support from family/friends was marginally related to healthful levels of light/moderate physical activity (OR 1.26, 95% CI [0.96, 1.65]) and television viewing (OR 1.29, 95% CI [0.99, 1.69]). Both sources of support were strongly associated with lower odds of incident depression (OR 0.33, 95% CI [0.20, 0.55] and OR 0.49, 95% CI [0.30, 0.79], respectively). We did not find associations with vigorous physical activity or sleep duration.

**Conclusions:**

Social support is important to the physical and mental health of new mothers and may promote behaviors that limit postpartum weight retention.

## Background

Postpartum weight retention (PPWR) is a major contributor to long-term obesity among some women. Substantial PPWR, often defined as retaining at least 5 kg between pre-pregnancy and 1-year postpartum, is experienced by up to 13% of women [1, 2]. Established risk factors for substantial PPWR include pre-pregnancy obesity and excessive gestational weight gain [3, 4]. It has been reported that postpartum factors that may buffer risk for PPWR include behaviors such as having adequate sleep, minimizing sedentary time, and consuming a healthful diet, whereas postpartum depression increases risk [5–7]. However, few studies have investigated psychosocial predictors of the factors that attenuate or increase the risk of PPWR.

Social support is one aspect of women’s life that may help new mothers adopt or maintain healthier behaviors that are linked to lower PPWR. Thus, it is not surprising that limited social support has been found to be related to depression in the postpartum period and that support from a partner, family or friends may help a new mother cope with stress and mental health issues [8, 9]. Social support may also help mothers engage in healthy behaviors, but the evidence is more sparse in postpartum period [10]. Very few studies of general populations have examined the relationships of PPWR, weight retention related health behaviors, and mood disorders with social support from partners and from family/friends concomitantly during the postpartum months [11, 12].

The aim of the current study was to explore the relationships of partner and family/friends social support at 6 months postpartum with women’s health behaviors and mental health that have previously been shown to predict postpartum weight retention (dietary intake of fiber and trans-fat, physical activity, sedentary behavior, sleep quality and depressive symptoms). The hypothesis was that higher levels of perceived social support from either a partner or family/friends would be associated with positive health behaviors among mothers at 6 months postpartum as well as with PPWR and weight change up to 1 year postpartum.

## Methods

Participants were women enrolled in Project Viva, a prospective observational cohort study of prenatal factors, pregnancy outcomes, and offspring health. Between 1999 and 2002, we recruited pregnant women from 8 obstetric offices of Atrius Harvard Vanguard Medical Associates, a multispecialty group practice in eastern Massachusetts. We approached women when attending their initial obstetric visit, which occurred at median 9.9 weeks of gestation. Research assistants met potential participants in the waiting room in order to introduce the study, and determined their eligibility. We excluded women with multiple gestation, who were not able to complete questionnaires in English, with a gestational age ≥ 22 weeks at enrollment, and women who anticipated moving away before delivery. All participating women provided written informed consent, and the institutional review board of Harvard Pilgrim Health Care reviewed and approved the project in line with ethical standards established by the Declaration of Helsinki.

Of the 2128 women who delivered a live infant, 93 disenrolled before the 6-month visit and 338 declined or were not reached at 6 months. Of the 1697 women with any data at the 6-month visit, 1443 completed a 6-month questionnaire. As shown in Additional File 1, the 1443 women who completed a 6-month questionnaire were slightly older [mean (SD) at enrollment 32.5 (4.7) vs 30.4 (5.9) years] and were more likely to be college graduates (73% vs. 48%) compared with the 685 women who did not complete a 6-month questionnaire. For this analysis, we excluded 41 participants who were missing data on social support or partner status and 46 who reported they did not have a partner. The final analytical sample was 1356 mothers. The analytic sample included women who were slightly older at enrollment [32.6 (4.6) vs. 30.4 (5.9) years] and were more likely to report being white (76% vs. 50%), be college graduates (74% vs. 48%) and have a household income greater than $70,000/year (68% vs. 46%) compared with the 772 excluded women.

### Exposures: social support

On a questionnaire completed at an in-person visit or via mail at 6 months postpartum, women completed the Turner Support Scale (previously published and validated) to assess their level of perceived partner and family/friends support [13]. Each question was answered using a 4-point Likert scale (ranging from *Strongly disagree* = 0 to *Strongly agree* = 3). The Turner Support Scale includes items about various types of social support including financial (for example: “I can count on my partner for financial assistance should I need it”) and emotional support (for example: “My family or friends let me know they think I am a worthwhile person”). Partner and family/friends support each had 5 items with response values coded as 0–3. For each of the two sources of support, we computed the sum of the 5 items (possible range 0–15), with higher scores indicating stronger support. The tool has been validated among pregnant women with excellent reliability (Cronbach’s alpha = .94) [13]. In the present study, the Cronbach’s alpha was .80 for the partner support scale and .92 for the family/friends support scale.

### Outcomes: obesogenic risk factors and postpartum weight retention

Outcomes of interest were selected based on behaviors previously shown to predict substantial PPWR in this cohort [3, 7]. At 6 months postpartum, women completed the PrimeScreen, a brief previously published and validated dietary survey that includes 21 questions about intake of foods and food groups [14]. The time referent was “since your baby was born”. To obtain estimates of nutrients, we used the Harvard nutrient composition database [15]. We chose to include dietary fiber and trans-fatty acid intake in this analysis based on previous research from our group where these two nutrients were associated with PPWR [5, 7]. We categorized healthier intake of fiber as ≥ median and trans-fat intake as < median.

In addition, participants reported the average weekly hours they spent watching television or videos and in leisure-time physical activity using questions derived from the previously published and validated Physical Activity Scale for the Elderly (PASE) [16]. Physical activity was classified as walking (“for fun or exercise, including to or from work, but not at work”), light/moderate physical activity (“such as yoga, bowling, stretching classes, and skating, not including walking”), and vigorous recreational activities (“such as jogging, swimming, cycling, aerobic class, skiing, or other similar activities”). We categorized healthier amounts as < 2 h per day watching television, ≥ 30 min per day walking, any light/moderate physical activity, and any vigorous physical activity. We combined light and moderate activities into one question that did not include walking [7]. Moreover, instead of using the previous 7 days as in the PASE questionnaire, women averaged their activity over the previous month, and reported average hours per week as used in previous studies [17]. Participants also reported the average number of hours of sleep they obtained during a 24-h period during the last month (healthier categorized as ≥ 6 h per day) [5]. These self-reported measures of health behaviors have been used in numerous studies and have high correlations with their respective objective measure [7, 14, 18]. Women self-reported their pre-pregnancy weight at enrollment and at 1 year postpartum on questionnaires. We calculated PPWR as the difference between 1 year postpartum and pre-pregnancy weight, in kg and defined substantial PPWR as ≥ 5 kg [5] and healthy PPWR as < 2 kg [19]. At 6 months postpartum, women completed the 10-item Edinburgh Postnatal Depression Scale (EPDS) as a measure of depressive symptoms [20, 21]. A score ≥ 13 on a 0–30 scale indicates probable depression. In this study, the internal consistency for EPDS was high with Cronbach’s alpha of .87.

### Covariates

We assessed information on participant demographics from Project Viva questionnaires and interviews. Mothers reported their age, race/ethnicity, parity, education, pregnancy smoking status, and household income. We categorized these characteristics as shown in Table 1. Specifically, mothers reported the highest level of education completed as follows: Less than 12th grade, High school degree or a GED, Some college or an associate degree, 4 years of college (BA, BS) or Graduate degree (Master’s, Ph.D.) and we dichotomized responses as ≥ college degree or not. Annual household income was categorized as $5000 or Less, 5001 to 10,000, 10,001 to 20,000, 20,001 to 40,000, 40,001 to 70,000, or More than 70,000 and we dichotomized responses as > $70,000 or not. We asked mothers at both first and second trimester visits about their cigarette smoking habits before and during pregnancy and categorized smoking as never, former, and smoked during pregnancy. We calculated maternal pre-pregnancy body mass index (BMI, kg/m^2^) using self-reported pre-pregnancy weight and height. We calculated gestational weight gain as the difference between the last clinically measured weight prior to delivery (within 4 weeks before delivery) and self-reported pre-pregnancy weight.

**Table 1:** Selected demographics, perceived social support and health behaviors at 6 months and 1 year postpartum among 1356 women in the Project Viva cohort

Participant demographics	Mean (SD) or %
Age (years)	32.6 (4.6)
Pre-pregnancy BMI (kg/m^2^)	24.5 (5.2)
Pregnancy weight gain (kg)	15.6 (5.4)
Race/ethnicity	
White	76%
Black	9%
Hispanic	6%
Asian	6%
Other	3%
Nulliparous	49%
College graduate	74%
Household income > $70,000/year	68%
Pregnancy smoking status	
Never	69%
Former	22%
During pregnancy	9%
Exposures at 6 months postpartum	
Partner support (points)	12.8 (2.3)
Family/friends support (points)	12.9 (2.5)
Outcomes at 6 months postpartum	
Walking ≥ 30 min/day	56%
Any light/moderate physical activity	40%
Any vigorous physical activity	35%
Fiber intake > median	50%
Trans-fat intake < median	50%
Sleep ≥ 6 h/day	88%
Watching television < 2 h/day	67%
Incident depression	6%
Outcomes at 1 year postpartum	
PPWR (kg)	0.5 (4.7)
PPWR categories	
< 2 kg	65%
2 to < 5 kg	23%
≥ 5 kg (SPPWR)	12%

*Note. PPWR* Postpartum weight retention, *SPPWR* Substantial postpartum weight retention

### Statistical analysis

We used a series of multivariable logistic regression models to evaluate associations of perceived social support (from partner and family/friends) with each of the outcome variables. Model 1 examined partner support alone, Model 2 examined family/friends support alone, and Model 3 included both sources of social support simultaneously in the analysis. In Model 4, we adjusted for characteristics that could be independent predictors of outcomes or might confound exposure-outcome associations, including age, race/ethnicity, parity, education, smoking during pregnancy, and household income as well as pre-pregnancy BMI and total gestational weight gain. We evaluated the associations of social support with outcomes using a 5-point increment because this was the interquartile range for family/friends social support. Using a multinomial logistic regression, we also examined associations of social support from partner and family/friends with 1-year PPWR categorized as 2 to < 5 kg and ≥ 5 kg and used < 2 kg as the comparison group. We excluded women with mid-pregnancy depression from analyses predicting incident postpartum depression and women who were currently pregnant at 1 year from analyses predicting 1-year PPWR.

We evaluated linearity using generalized additive models with smoothing terms for partner support and family/friends support, and we confirmed that the associations did not depart from linearity (*p*-values > 0.05). Using Hosmer-Lemeshow Goodness-of-Fit Tests, we observed good model fit (all *p*-values > 0.05). We used dichotomous outcomes thus reducing the impact of outliers. Partner and family/friends support and some covariates were correlated but not collinear. We performed all analyses using SAS version 9.4 (SAS Institute Inc., Cary, NC).

## Results

### Participants

Mean (SD) age at study enrollment was 32.6 years (4.6), pre-pregnancy BMI was 24.5 kg/m^2^ (5.2) and total gestational weight gain was 15.6 kg (5.4); 76% of women were white and 74% were college graduates (Table 1). The mean (SD) partner support score was 12.8 (2.3) points, the median was 13 and the interquartile range was 11–15. The mean (SD) family/friends support was 12.9 (2.5) points, the median was 14 and the interquartile range was 10–15. Both partner support and family/friends support scores range from 0 to 15. Distributions of partner and family/friends questions are presented in Table 2. Partner support and family/friends support were correlated with each other (Pearson *r* = .42, *p* < .0001). At 6 months postpartum, 56% of the women walked ≥ 30 min/d, 40% engaged in any light/moderate physical activity (other than walking), 35% engaged in any vigorous physical activity, 88% slept ≥ 6 h/d, and 67% watched television < 2 h/d. Among women who had not reported high depressive symptoms during pregnancy, 6% reported incident probable depression (EPDS score ≥ 13) at 6 months postpartum. At 1 year, mean (SD) PPWR was 0.5 kg (4.7); 65% of women had PPWR < 2 kg, 23% 2 to < 5 kg, and 12% ≥ 5 kg.

**Table 2:** Distributions of partner and family/friends social support scale questions among 1356 women in the Project Viva cohort

	Strongly disagree	Disagree	Agree	Strongly agree
Support scale questions	%			
Partner				
"I can count on my partner for financial assistance should I need it.”	1%	3%	14%	82%
"My partner is affectionate toward me.”	1%	3%	26%	70%
"My partner helps a lot with the baby.”	1%	5%	33%	60%
"My partner understands how I am feeling.”	1%	13%	53%	33%
"I can count on my partner to be there when I need him/her.”	< 1%	3%	29%	68%
Family/friends				
"My family or friends let me know they think I am a worthwhile person.”	< 1%	2%	36%	61%
"My family or friends have confidence in me.”	< 1%	1%	32%	66%
"My family or friends provide me with help in finding solutions to my problems.”	< 1%	5%	40%	55%
"My family or friends will always stand by me.”	< 1%	1%	30%	68%
"I can count on my family or friends for financial assistance should I need it.”	2%	6%	34%	58%

### Perceived social support from partner

In unadjusted logistic regression models, each 5*-*point increment in perceived support from a partner was associated with higher odds of walking for at least 30 min per day (OR 1.34; 95% CI [1.06, 1.71]), as well as higher odds of consuming above the median amount of fiber (OR 1.61; 95% CI [1.26, 2.05]) and below the median amount of trans-fat (OR 1.88; 95% CI [1.47, 2.41]) (Table 3, Model 1)*.* Perceived support from a partner was also associated with lower odds of incident depression (OR 0.23; 95% CI [0.15, 0.35]). We did not find significant associations between perceived partner support and light/moderate physical activity (OR 1.22; 95% CI [0.96, 1.56]), vigorous physical activity (OR 1.13; 95% CI [0.88, 1.46]), sleep duration (OR 1.37; 95% CI [0.97, 1.93]) or television viewing (OR 0.95; 95% CI [0.74, 1.22]) (Table 3). This pattern of results was similar in models additionally adjusted for family/friends support (see Model 3) and after the inclusion of all covariates in the analyses (Model 4 in Table 3). Each 5-point increment of partner social support was associated with lower odds of PPWR 2 to < 5 kg (OR 0.70; 95% CI [0.50, 0.98]) and lower odds of PPWR ≥ 5 kg (OR 0.63; 95% CI [0.41, 0.96]), compared to PPWR < 2 kg in Model 1; the estimates were slightly attenuated with wider confidence intervals crossing 1 in fully adjusted Model 4 (Table 3).

**Table 3:** Associations of partner and family/friends support with beneficial postpartum behaviors and depression at 6 months and 1 year postpartum among 1356 women in the Project Viva cohort

Outcome	Exposure (per 5 points)	Model 1 Partner support	Model 2 Family/friends support	Model 3 Combined Partner and Family/friends support	Model 4 Model 3 + Covariates
		OR [95% CI] per 5 Points Social Support			
6 months postpartum					
Walking ≥ 30 min/d	Partner support	1.34 [1.06, 1.71]		1.31 [1.00, 1.70]	1.36 [1.01, 1.82]
	Family/friends support		1.18 [0.95, 1.46]	1.07 [0.84, 1.35]	1.23 [0.95, 1.59]
Light/moderate PA any	Partner support	1.22 [0.96, 1.56]		1.07 [0.82, 1.41]	0.98 [0.73, 1.31]
	Family/friends support		1.36 [1.09, 1.70]	1.33 [1.04, 1.70]	1.26 [0.96, 1.65]
Vigorous PA any	Partner support	1.13 [0.88, 1.46]		1.11 [0.85, 1.47]	1.02 [0.75, 1.39]
	Family/friends support		1.08 [0.86, 1.35]	1.04 [0.81, 1.33]	0.92 [0.70, 1.21]
Fiber ≥ median	Partner support	1.61 [1.26, 2.05]		1.50 [1.15, 1.96]	1.43 [1.06, 1.91]
	Family/friends support		1.35 [1.09, 1.67]	1.16 [0.91, 1.47]	1.12 [0.86, 1.45]
Trans-fat < median	Partner support	1.88 [1.47, 2.41]		1.72 [1.31, 2.25]	1.49 [1.11, 2.01]
	Family/friends support		1.49 [1.20, 1.85]	1.22 [0.96, 1.55]	1.11 [0.85, 1.44]
Sleep ≥ 6 h/d	Partner support	1.37 [0.97, 1.93]		1.16 [0.79, 1.69]	1.05 [0.70, 1.59]
	Family/friends support		1.52 [1.12, 2.07]	1.44 [1.02, 2.03]	1.17 [0.81, 1.71]
Television < 2 h/d	Partner support	0.95 [0.74, 1.22]		0.85 [0.64, 1.12]	0.77 [0.57, 1.05]
	Family/friends support		1.20 [0.96, 1.50]	1.28 [1.00, 1.63]	1.29 [0.99, 1.69]
Incident depression	Partner support	0.23 [0.15, 0.35]		0.32 [0.20, 0.50]	0.33 [0.20, 0.55]
	Family/friends support		0.31 [0.21, 0.46]	0.45 [0.29, 0.70]	0.49 [0.30, 0.79]
1 year postpartum (compared to PPWR < 2 kg)					
PPWR 2 to < 5 kg	Partner support	0.70 [0.50, 0.98]		0.73 [0.50, 1.07]	0.76 [0.50, 1.14]
	Family/friends support		0.79 [0.58, 1.09]	0.89 [0.63, 1.27]	1.05 [0.72, 1.55]
SPPWR ≥ 5 kg	Partner support	0.63 [0.41, 0.96]		0.65 [0.41, 1.03]	0.75 [0.43, 1.32]
	Family/friends support		0.79 [0.52, 1.19]	0.93 [0.59, 1.46]	1.13 [0.66, 1.93]

*Note. OR* Odd ratio, *CI* Confidence interval, *PA* Physical activity, *SPPWR* Substantial postpartum weight retention (≥ 5 kg)

Model 1: Partner support only; Model 2: Family/friends support only; Model 3: Partner support and family/friends support;

Model 4: Model 3 + age, race/ethnicity, parity, education, smoking during pregnancy, household income, pre-pregnancy BMI and

gestational weight gain

### Perceived social support from family/friends

Each 5-point increment in perceived support from family and friends was associated with higher odds of any light/moderate physical activity (OR 1.36; 95% CI [1.09, 1.70]) and sleep duration ≥ 6 h/day (OR 1.52; 95% CI [1.12, 2.07]) (Table 3, Model 2). Similar to the findings for partner social support, support from family and friends was associated with higher odds of consuming above the median amount of fiber (OR 1.35; 95% CI [1.09, 1.67]), below the median amount of trans-fat (OR 1.49; 95% CI [1.20, 1.85]) and lower odds of incident probable depression (OR 0.31; 95% CI [0.21, 0.46]). Adding covariates attenuated the strength of associations with assessed behaviors (Model 4 in Table 3) and only the association with probable depression the 95% CI of our estimate did not cross 1. We did not observe associations of family/friends support with PPWR categories (Table 3).

## Discussion

The aim of the current study was to explore the protective role of perceived social support, from a partner and/or from family/friends, in helping new mothers engage in healthful lifestyle behaviors and buffering against postpartum depression, factors that would reduce the risk of PPWR. As hypothesized, greater perceived social support was associated with many of the evaluated components of physical and mental health. Specifically, higher partner support was associated with higher likelihood of walking regularly, healthful diet habits, while social support from family/friends seemed to be associated with engaging in physical activity of light/moderate intensity. Additionally, women who felt supported by either their partner or family/friends member were less likely to experience depressive symptoms in the postpartum period.

Phillips, King and Skouteris have developed a conceptual model that acknowledges the role of psychological predictors of PPWR and proposed that socio-contextual factors, physiological factors, psychological distress and health behaviors all contribute to maternal weight post-delivery [22]. Indeed, PPWR is known to be associated with a myriad of factors contributing to postpartum physical and mental health [23–25]. It appears from our findings that perceived social support matters in terms of helping mothers to be physically and mentally healthy in the postpartum period and that partner and family/friends may both have influence. In fact, few studies have examined relationships among health behaviors, mood disorders and social support in the stressful period following child birth. Using a large cohort, we have shown that both partner and family/friends may help to prevent depressive symptoms, but partner support specifically may promote healthful behaviors like walking regularly and maintaining a healthful diet.

In particular, the partner may have a protective role by supporting the mother in her new role and enable her to cope with physical or mental health issues in the postpartum period [26]. These current results suggest that partners may help the new mother in activities of daily living, providing support on health behaviors that occur every day like healthful food intake as well as instrumental support or company to engage in daily walking. Others have shown that social support is likely to be associated with higher self-efficacy [27], the feeling of having the capacity to achieve goals or to cope with unexpected situations, which may help women maintain their healthy choices by positively influencing their intention and behaviors across time [28]. These daily positive choices in turn seem to lead to lower weight retention 1 year after delivery [7]. All these findings support the critical role partner support plays in the health of new mothers in the postpartum period.

Postpartum depression may potentially affect the whole family and is likely to be associated with unhealthy behaviors [29, 30]. Our findings support that both partner and family/friends support have a positive effect on women’s mental health in the postpartum period. Therefore, these finding that mothers with higher perceived social support from their partner and/or their family/friends were less likely to experience depressive symptoms might be due to emotional social support providing a coping strategy to combat depressive symptoms [31]. In fact, depression risk may be reduced when social support offers emotional and social resources to help the women coping with stress and adjust to motherhood [31]. Our finding further strengthens the known inverse association between social support and depression [8], as well as identifies that multiple people in a new mother’s life can provide them with such support.

Family and friends may also provide new mothers with the practical support needed to engage in physical activity of light/moderate intensity. In contrast to daily walking, which most women can do together with their baby, this type of activity may happen less frequently as it often requires time apart from baby. We speculate that family and friends may provide company to encourage such activity or also may offer child care to facilitate these activities, allowing mothers to have time to maintain healthy behaviors [32]. Yet, the strength of association we observed between family/friends support and physical activity was reduced and the wide CI included 1 after adjusting for potential confounders, thus it is possible that this association is partially explained by other factors.

The findings from the current study need to be interpreted within the context of some methodological limitations. While this cohort is well characterized with information on many important covariates including pre-pregnancy BMI and gestational weight gain, the sample contains a relatively high proportion of women who were college graduates, white and from higher-income households, thereby results may not generalize to women of other socioeconomic or ethnic backgrounds. Thus, future studies would benefit from investigating the extent to which the associations observed in the current study are also found in minority or less privileged populations. Health outcomes used in this study are self-reported and subject to recall and social desirability bias. Depressive symptoms in particular may be over or under estimated given the small numbers of items of the EPDS. Moreover, the PrimeScreen was not designed to assess total diet, and therefore does not measure total energy intake. This factor limits our ability to estimate absolute nutrient intakes and compare them with existing standards, such as recommended daily allowances for dietary fiber and trans-fat. Moreover, although the PrimeScreen was validated against both a longer food frequency questionnaire and biomarkers among non-pregnant adults [14], it may not fully represent dietary intake in the first 6 months postpartum. Also, screen time including phone, tablets and computers, was not included in this study but was probably more limited in use in the early 2000s. We did not directly measure intensity of walking and physical activity. Future studies might consider incorporating objective measures of behaviors such as accelerometry, although these methods are generally more intrusive and expensive and may not be feasible in all settings. We controlled for smoking status during pregnancy, however we did not control for smoking during the postpartum period. Given the fact that the majority of women in this cohort reported high levels of support, the lack of variability in the social support measure may also have reduced our ability to detect significant associations. Since our design is observational, causality cannot be determined. Finally, given the number of associations examined, we remained prudent about interpretation of statistical significance for any single model; but rather focused on patterns of associations across different models and outcomes.

## Conclusions

The current study highlights the potential protective role of perceived social support to have a positive impact across a range of physical behaviors and mental health factors, which in turn are known to influence PPWR. More specifically, partner support was associated with greater likelihood of frequent walking, greater fiber intake, and lower intake of trans-fat, while support from family/friends showed a tendency towards higher participation in light/moderate physical activity. Both sources of social support were associated with lower incidence of probable postpartum depression. As such, enhancing social support amongst new mothers may contribute to obesity prevention efforts, as it can improve both behaviors and mental health symptoms associated with risk for postpartum weight retention.

## Supplementary information

**Additional file 1:** **Table S1.** Participant characteristics overall and according to 6-month questionnaire completion status among 2128 women participating in Project Viva.

## Data Availability

The datasets used and/or analyzed during the current study are available from the corresponding author on reasonable request.

## Abbreviations

BMI: Body Mass Index
EPDS: Edinburgh Postnatal Depression Scale
PPWR: Postpartum Weight Retention

## References

[1] Rode L, Kjærgaard H, Ottesen B, Damm P, Hegaard HK (2012). Association between gestational weight gain according to body mass index and postpartum weight in a large cohort of Danish women. Matern Child Health J.

[2] Sumithran P, Houlihan C, Shub A, Churilov L, Pritchard N, Price S (2018). How common is substantial weight gain after pregnancy?. Obes Res Clin Pract.

[3] Oken E, Kleinman KP, Belfort MB, Hammitt JK, Gillman MW (2009). Associations of gestational weight gain with short- and longer-term maternal and child health outcomes. Am J Epidemiol.

[4] Rong K, Yu K, Han X, Szeto IM, Qin X, Wang J (2014). Pre-pregnancy BMI, gestational weight gain and postpartum weight retention: a meta-analysis of observational studies. Public Health Nutr.

[5] Gunderson EP, Rifas-Shiman SL, Oken E, Rich-Edwards JW, Kleinman KP, Taveras EM (2008). Association of fewer hours of sleep at 6 months postpartum with substantial weight retention at 1 year postpartum. Am J Epidemiol.

[6] Herring SJ, Rich-Edwards JW, Oken E, Rifas-Shiman SL, Kleinman KP, Gillman MW (2008). Association of postpartum depression with weight retention 1 year after childbirth. Obesity..

[7] Oken E, Taveras EM, Popoola FA, Rich-Edwards JW, Gillman MW (2007). Television, walking, and diet - associations with postpartum weight retention. Am J Prev Med.

[8] Cruise SM, Layte R, Stevenson M, O’Reilly D. Prevalence and factors associated with depression and depression-related healthcare access in mothers of 9-month-old infants in the Republic of Ireland. Epidemiol Psychiatr Sci. 2017;27(6):468-78. 10.1017/S2045796017000026 PMC6999010 28196546

[9] Gremigni P, Mariani L, Marracino V, Tranquilli AL, Turi A (2011). Partner support and postpartum depressive symptoms. J Psychosom Obstet Gynecol.

[10] Graham M, Uesugi K, Olson C (2016). Barriers to weight-related health behaviours: a qualitative comparison of the socioecological conditions between pregnant and post-partum low-income women. Matern Child Nutr.

[11] Saxbe DE, Schetter CD, Guardino CM, Ramey SL, Shalowitz MU, Thorp J (2016). Sleep quality predicts persistence of parental postpartum depressive symptoms and transmission of depressive symptoms from mothers to fathers. Ann Behav Med.

[12] Nagl M, Linde K, Stepan H, Kersting A (2015). Obesity and anxiety during pregnancy and postpartum : a systematic review. J Affect Disord.

[13] Turner RJ, Grindstaff CF, Phillips N (1990). Social support and outcome in teenage pregnancy. J Health Soc Behav.

[14] Rifas-Shiman SL, Willett WC, Lobb R, Kotch J, Dart C, Gillman MW (2001). PrimeScreen, a brief dietary screening tool: reproducibility and comparability with both a longer food frequency questionnaire and biomarkers. Public Health Nutr.

[15] Rimm EB, Giovannucci EL, Stampfer MJ, Colditz GA, Litin LB, Willett WC (1992). Reproducibility and validity of an expanded self-administered semiquantitative food frequency questionnaire among male health professionals. Am J Epidemiol.

[16] Washburn RA, McAuley E, Katula J, Mihalko SL, Boileau RA (1999). The physical activity scale for the elderly (PASE): evidence for validity. J Clin Epidemiol.

[17] Stuebe AM, Oken E, Gillman MW (2009). Associations of diet and physical activity during pregnancy with risk for excessive gestational weight gain. Am J Obstet Gynecol.

[18] Ekelund U, Sepp H, Brage S, Becker W, Jakes R, Hennings M (2006). Criterion-related validity of the last 7-day, short form of the international physical activity questionnaire in Swedish adults. Public Health Nutr.

[19] Cedergren MI (2007). Optimal gestational weight gain for body mass index categories. Obstet Gynecol.

[20] Cox JL, Holden JM, Sagovsky R (1987). Detection of postnatal depression. Development of the 10-item Edinburgh postnatal depression scale. Br J Psychiatry.

[21] Evans J, Heron J, Francomb H, Oke S, Golding J (2001). Cohort study of depressed mood during pregnancy and after childbirth. Br Med J.

[22] Phillips J, King R, Skouteris H (2012). A conceptual model of psychological predictors of postpartum weight retention. J Reprod Infant Psychol.

[23] Althuizen E, van Poppel MNM, de Vries JH, Seidell JC, van Mechelen W (2011). Postpartum behaviour as predictor of weight change from before pregnancy to one year postpartum. BMC Public Health.

[24] Goldstein ND, Rogers S, Ehrenthal DB (2016). The impact of psychosocial stressors on postpartum weight retention. Arch Womens Ment Health.

[25] Zanotti J, Capp E, Wender MCO (2015). Factors associated with postpartum weight retention in a Brazilian cohort. Rev Bras Ginecol E Obstet.

[26] Reid KM, Taylor MG (2015). Social support, stress, and maternal postpartum depression: a comparison of supportive relationships. Soc Sci Res.

[27] Karademas EC (2006). Self-efficacy, social support and well-being: the mediating role of optimism. Personal Individ Differ.

[28] Sheeran P, Maki A, Montanaro E, Avishai-Yitshak A, Bryan A, Klein WMP (2016). The impact of changing attitudes, norms, and self-efficacy on health-related intentions and behavior: a meta-analysis. Health Psychol.

[29] Letourneau NL, Dennis C-L, Benzies K, Duffett-Leger L, Stewart M, Tryphonopoulos PD (2012). Postpartum depression is a family affair: addressing the impact on mothers, fathers, and children. Issues Ment Health Nurs.

[30] Dubber S, Reck C, Müller M, Gawlik S (2015). Postpartum bonding: the role of perinatal depression, anxiety and maternal–fetal bonding during pregnancy. Arch Womens Ment Health.

[31] Li Y, Long Z, Cao D, Cao F (2017). Social support and depression across the perinatal period: a longitudinal study. J Clin Nurs.

[32] Kaiser B, Jeannot E, Razurel C (2016). Determinants of health behaviors after gestational diabetes mellitus: a prospective cohort study in Geneva. J Midwifery Womens Health.

