# Supplementary material for: Women’s perceived social support: associations with postpartum weight retention, health behaviors and depressive symptoms
Source: BMC Womens Health. 2019 Nov 21;19:143. doi: 10.1186/s12905-019-0839-6 (PMC6873672; doi:10.1186/s12905-019-0839-6)
Supplement: Supplementary file 1 — Additional file 1: Table S1. Participant characteristics overall and according to 6-month questionnaire completion status among 2128 women participating in Project Viva. [file 12905_2019_839_MOESM1_ESM.docx]

Additional file 1: Table S1

Participant characteristics overall and according to 6-month questionnaire completion status among 2,128 women participating in Project Viva.

|  |  | **Completed a 6-Month Questionnaire** | |
| --- | --- | --- | --- |
| **Characteristics** | **Overall** | **Yes** | **No** |
|  | *n* = 2,128 | *n* = 1,443 | *n* = 685 |
|  | **Mean (SD)** | | |
| Age (years) | 31.8 (5.2) | 32.5 (4.7) | 30.4 (5.9) |
| Pre-pregnancy BMI (kg/m^2^) | 24.9 (5.5) | 24.5 (5.2) | 25.6 (6.0) |
| Pregnancy weight gain (kg) | 15.5 (5.7) | 15.6 (5.5) | 15.4 (6.1) |
|  | ***N* (%)** | | |
| Race/ethnicity |  |  |  |
| White | 1,399 (66.5) | 1,068 (74.4) | 331 (49.6) |
| Black | 348 (16.5) | 152 (10.6) | 196 (29.3) |
| Hispanic | 154 (7.3) | 85 (5.9) | 69 (10.3) |
| Asian | 120 (5.7) | 82 (5.7) | 38 (5.7) |
| Other | 83 (3.9) | 49 (3.4) | 34 (5.1) |
| Nulliparous |  |  |  |
| No | 1,111 (52.2) | 728 (50.5) | 383 (55.9) |
| Yes | 1,017 (47.8) | 715 (49.5) | 302 (44.1) |
| College graduate |  |  |  |
| No | 744 (35.4) | 393 (27.4) | 351 (52.5) |
| Yes | 1,360 (64.6) | 1,043 (72.6) | 317 (47.5) |
| Household income > $70,000/year | |  |  |
| No | 728 (38.8) | 434 (32.4) | 294 (54.9) |
| Yes | 1,146 (61.2) | 904 (67.6) | 242 (45.1) |
| Pregnancy smoking status | |  |  |
| Never | 1,443 (68.5) | 993 (69.1) | 450 (67.2) |
| Former | 398 (18.9) | 305 (21.2) | 93 (13.9) |
| During pregnancy | 266 (12.6) | 139 (9.7) | 127 (19.0) |
